# Generalized Representation of Stereoscopic Surface Shape and Orientation in the Human Visual Cortex

**DOI:** 10.3389/fnhum.2019.00283

**Published:** 2019-08-20

**Authors:** Zhen Li, Hiroaki Shigemasu

**Affiliations:** ^1^Graduate School of Engineering, Kochi University of Technology, Kochi, Japan; ^2^School of Information, Kochi University of Technology, Kochi, Japan

**Keywords:** binocular vision, shape representation, orientation representation, fMRI, MVPA

## Abstract

The brain’s ability to extract three-dimensional (3D) shape and orientation information from viewed objects is vital in daily life. Stereoscopic 3D surface perception relies on binocular disparity. Neurons selective to binocular disparity are widely distributed among visual areas, but the manner in these areas are involved in stereoscopic 3D surface representation is unclear. To address this, participants were instructed to observe random dot stereograms (RDS) depicting convex and concave curved surfaces and the blood oxygenation level-dependent (BOLD) signal of visual cortices was recorded. Two surface types were: (i) horizontally positioned surfaces defined by shear disparity; and (ii) vertically positioned surfaces defined by compression disparity. The surfaces were presented at different depth positions per trial. Functional magnetic resonance imaging (fMRI) data were classified from early visual areas to higher visual areas. We determined whether cortical areas were selective to shape and orientation by assessing same-type stimuli classification accuracies based on multi-voxel activity patterns per area. To identify whether some areas were related to a more generalized sign of curvature or orientation representation, transfer classification was used by training classifiers on one dataset type and testing classifiers on another type. Same-type stimuli classification results showed that most selected visual areas were selective to shape and all were selective to the orientation of disparity-defined 3D surfaces. Transfer classification results showed that in the dorsal visual area V3A, classification accuracies for the discriminate sign of surface curvature were higher than the baseline of statistical significance for all types of classifications, demonstrating that V3A is related to generalized shape representation. Classification accuracies for discriminating horizontal–vertical surfaces in higher dorsal areas V3A and V7 and ventral area lateral occipital complex (LOC) as well as in some areas of intraparietal sulcus (IPS) were higher than the baseline of statistical significance, indicating their relation to the generalized representation of 3D surface orientation.

## Introduction

The ability to interact with objects in the real world is closely related to three-dimensional (3D) perception. This skill depends on at least two abilities as follows: (i) the ability to perceive the shape of a 3D object; and (ii) the ability to judge the orientation of the object. For example, when someone attempts to pick up a pencil on a desk or insert a key into a lock, the procedure depends on the above-mentioned abilities. Although these activities are common and essential in human daily life, their underlying visual mechanisms have not yet been completely investigated. Binocular disparity, which is generated by the horizontal separation of the two eyes, is one of the most important cues for 3D perception. It is an extremely informative cue that is sufficient for depicting any 3D percept imaginable—the depth of the points of the object as well as the surface shape and orientation, which are considered higher-order surface properties.

To extract useful information from images registered by the two eyes, a sequence of processing stages is required (Marr and Poggio, 1976). Different brain regions may play different roles in 3D shape/orientation processing, with some areas responsible for low-level disparity and other areas responsible for middle or higher stage 3D representations that do not depend on low-level disparity.

Previous studies of 3D perception from disparity have focused on the neurons’ selectivity to disparity depicting 3D objects. Zero-order depth indicates the depth position of an object. First-order depth corresponds to a linear gradient of depth, such as a plane slanted in depth. Second-order depth refers to curvature in depth, e.g., disparity curvature in stereoscopic processing (Orban et al., 2006b). In monkey and human brains, the neurons selective to binocular disparity exist at multiple levels of the visual hierarchy, starting from the early visual areas to the object-selective and motion-selective areas and parietal areas (Cumming and DeAngelis, 2001; Neri, 2005; Chandrasekaran et al., 2007; Parker, 2007). There is evidence that neurons tuned for 3D shape are widely distributed across the visual areas of the cortex. Janssen et al. (1999) verified that a portion of the inferotemporal (IT) neurons in macaques is selective for 3D shape depicted by binocular disparity. Janssen et al. (2001) conducted a single-cell method on animals and found that neurons of the lower bank of the superior temporal sulcus (TEs) can be selective for horizontal 3D shapes. Georgieva et al. (2009) tested the interaction between stereo and order of disparity, concluding that the V3A complex and certain intraparietal sulcus (IPS) regions can extract and process 3D shape from stereo. Recently, Alizadeh et al. (2018) studied a patch of the macaque posterior inferotemporal area TEO that is activated more by a curved surface than a flat surface. Using the single-cell method, they observed that this patch did not contain a large number of higher-order disparity-selective neurons; however, the sign of the disparity gradient of the stimuli could reliably be classified using a linear support vector machine (SVM). Orientation discrimination research has primarily focused on two types of orientation as follows: (i) tilt, which refers to rotation in a 2D image plane, similar to a clock hand changing its orientation over time; and (ii) slant, which refers to rotation toward/away from the frontoparallel direction. In relation to selectivity to orientation, Nguyenkim and DeAngelis (2003) found that several middle temporal area (MT) neurons in rhesus monkeys are tuned to 3D surface orientation defined by binocular disparity, and that tilt and slant typically exhibit independent effects on MT responses. Rosenberg et al. (2013) reported that in the caudal intraparietal area (CIP) of macaques, an explicit representation of slant exists, indicating that this area plays an important role in encoding surface orientation information. In functional imaging studies of humans, Shikata et al. (2001) found that both posterior (CIP) and anterior (AIP) areas within the IPS responded to planes oriented in depth using texture gradients as monocular cues. In addition, Naganuma et al. (2005) found that orientation information defined by disparity is processed in the parietal area. Ban and Welchman (2015) revealed a dorsal hierarchy that extracts 3D surface orientation from binocular disparity, demonstrating that responses in the V3A parallel the perceptual judgments of slant in humans.

Previous studies for shape/orientation representation of 3D object focused mainly on these types of questions: (1) selectivity of neurons to specific depth cues (e.g., binocular disparity) using single-cell studies; (2) blood oxygenation level-dependent (BOLD) signal changes to specific stimuli (e.g., 3D shape) vs. control condition using functional magnetic resonance imaging (fMRI) studies; (3) distinguishing BOLD signal patterns caused by different shapes/orientations; and (4) integration of different cues that concurrently describe a 3D object. Our present study belongs to the third type. However, to the best of our knowledge, previous studies have not investigated whether the shape/orientation presentation of stereopsis is directly dependent on disparity or to a more generalized processing. We investigated whether a generalized representation is involved by transfer classification of BOLD signal patterns. In detail, we investigated the sign of curvature (convex–concave shape) representation and horizontal–vertical orientation representation of the surface across cortical visual areas. The main purpose of our study was to identify the areas containing a reliable representation of the sign of curvature and orientation of the 3D surface and to determine the extent of generalization of the representation. Depending on the degree of reliability of the representation, the term “generalized representation of the sign of curvature” can be defined as follows: the lowest level is representation based on the local disparity pattern of stimuli (i.e., not generalized); the subsequent is representation based on a more global sign of curvature information, irrespective of the orientation of surfaces; and the more general level is representation based on the global information of the sign of curvature, irrespective of both the orientation of surfaces and depth position. Likewise, the term “generalized representation of the orientation of 3D surface” can be defined as follows: the lowest level is representation based on the local disparity pattern of stimuli; the next level is representation based on more global information, irrespective of the sign of curvature; the subsequent level is representation based on global information, irrespective of the sign of curvature and depth position. In our experiment, random dot stereograms (RDS) were used to depict horizontally or vertically positioned convex and concave stereoscopic surfaces; the sign of curvature and orientation of these stimuli cannot be judged by monocular cues but can be judged by binocular cues. The BOLD signal, as measured by fMRI, can be used to indirectly reflect the underlying neural activity of cortices (Logothetis et al., 2001). We tested the global BOLD signal patterns of each area by classification using multi-voxel pattern analysis (MVPA). The overall plan of the study was the following. First, the whether multi-voxel pattern is related to sign of curvature and orientation of surface depicted by binocular disparity in each area was assessed. Second, by using stimuli of the same sign of curvature (plus or minus) or orientation (horizontal or vertical) defined by different disparities, it was compared whether BOLD signal patterns invoked by stimuli with different disparities and the same sign of curvature/orientation are similar. Correspondingly, in the experiment, we initially sought to identify the areas involved in representing the sign of curvature and horizontal–vertical orientation of stereoscopic surfaces defined by disparity. We investigated the retinotopic visual cortices (V1, V2, V3d, V3v, V3A), the higher ventral cortex [lateral occipital complex (LOC)], the higher dorsal area [human middle temporal complex (hMT+), kinetic occipital area (KO), V7], and the IPS areas [the ventral intraparietal sulcus (VIPS), parieto-occipital intraparietal sulcus (POIPS), and dorsal intraparietal sulcus (DIPS)]. Because areas that are related to binocular disparity processing exist at the multiple levels of the visual hierarchy, we expected that high accuracies would be shown in most regions of interest (ROIs) for same-type stimuli classification. Furthermore, we tested whether the high accuracies relate to disparity information *per se* or to a more generalized processing independent of low-level retinotopic disparity information using transfer-type stimuli classification. We hypothesized that some middle or higher areas are involved in a more generalized representation of the sign of curvature, particularly in the dorsal areas V3A and KO. This could be attributed to the belief that the 3D visual information is progressively processed along the streams, with the early areas related to simple attribute processing and the middle and higher areas related to more complex attributes. It is possible that generalized representations exist in some middle and higher areas. Further, coarse stereopsis is processed in the dorsal stream and fine stereopsis is processed in the ventral stream (Schiller et al., 1990; Neri et al., 2004; Uka and DeAngelis, 2006; Roe et al., 2007). Coarse stereopsis can be used to guide the vergence movements of the eyes. During this process, it is possible that some abstract information regarding the shape is required and 3D shape is represented in a more abstract (coarse) way in the dorsal stream. Furthermore, 3D information is exchanged between the two streams and a more generalized representation of shape that can be shared between the dorsal and ventral streams may be involved. Van Dromme et al. (2016) provided causal evidence for the flow of visual 3D information between the dorsal stream and the ventral streams. V3A and KO are considered to be parts of the anatomical pathway where the functionally different ventral and dorsal streams exchange information (Takemura et al., 2016). In addition, Dövencioğlu et al. (2013) demonstrated that KO plays an important role in integrating disparity and shading cues of 3D structure perception, suggesting a more generalized representation of depth structure in the dorsal stream. Therefore, V3A and KO are two of the candidate areas for generalized sign of curvature representation. For orientation representation, IPS areas are expected to be involved in the more generalized representations of horizontal–vertical orientation because previous studies have shown that the neurons in the posterior IPS areas in monkeys and humans are selective to orientation *via* monocular cues, such as texture gradients and perspective, as well as *via* binocular cues, such as binocular disparity (Shikata et al., 2001; Tsutsui et al., 2001; Naganuma et al., 2005; Rosenberg et al., 2013).

## Materials and Methods

### Participants

A total of 11 participants were recruited for the fMRI experiments. Of these, three participants were excluded due to poor performance on judging convex and concave stereoscopic surfaces. “Acceptable performance” was defined as an accuracy of ≥75% for each stimulus type. Of the remaining eight participants, seven were male and one was female. One of the males was left-handed, whereas the other seven participants were right-handed. All participants had normal or corrected-to-normal vision. None had any history of mental illness or neurological disease. Their ages ranged from 22 to 33 (mean ± SD, 24.6 ± 3.7) years. Participants were remunerated for their participation.

This study was performed in accordance with the recommendations of Human Research Ethics Committee of the Kochi University of Technology, and written informed consent was obtained from all participants. All subjects gave written informed consent in accordance with the Declaration of Helsinki. The protocol was approved by the Human Research Ethics Committee of the Kochi University of Technology.

### Stimuli

Stereoscopic stimuli were presented on a screen using a pair of JVC D-ILA video projectors. A linear polarized filter was placed in front of each projector. The images from the two projectors were superimposed into one image and projected onto a translucent screen inside the bore of the magnet. The participants wore polarized glasses and viewed stimuli through a slanted mirror (angled at 45°) above the head coil. The optical distance from the midpoints of the two eyes to the screen was 71 cm. The screen resolution was set at 1,024 × 768 pixels and the refresh rate was 120 Hz.

**Figure 1:**
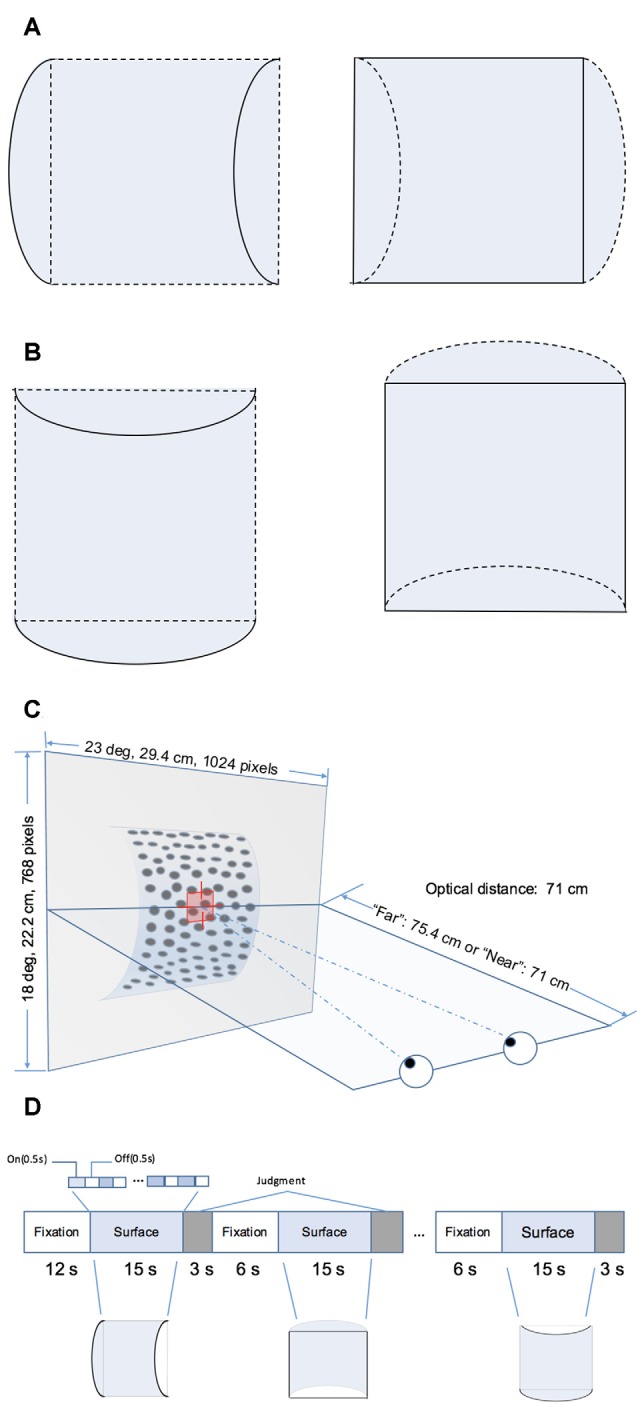
Schematic illustration of the stimuli and block design. **(A)** Horizontal hemi-cylindrical convex (left) and concave (right) surfaces. **(B)** Vertical hemi-cylindrical convex (left) and concave (right) surfaces. **(C)** Experimental set up (the example is a convex near surface). **(D)** Illusion of the functional magnetic resonance imaging (fMRI) block design.

The RDS stimuli comprised random black and white dots generated using Psychotoolbox 3 in MATLAB (The MathWorks, Natick, MA, USA). They depicted four surface types defined by disparity as follows: the horizontal convex and concave curved surfaces (Figure 1A) and vertical convex and concave curved surfaces (Figure 1B). Each surface covered a square of 14° × 14° and was presented on a mid-gray rectangular background (23° × 18°). The density of the stereogram was 41 dots/deg^2^; all the dots were of the same size and the diameter of the dots was 0.14°. The radius of the surface was 4.4 cm. The horizontal disparity of dots was calculated based on the depth position and there was no vertical disparity between corresponding dots. A fixation marker comprising a hollow square with a side length of 0.5° and horizontal and vertical nonius lines with a length of 0.375° was displayed at the center of the screen to help participants maintain eye vergence. Figure 1C shows the experimental set-up.

### Experimental Design

Four stimulus types, namely horizontal convex, horizontal concave, vertical convex, and vertical concave hemi-cylindrical surfaces, were presented during the experiment at two different depth positions (“near” or “far”) behind the fixation marker. The distance from the nearest part of the surface and fixation marker was simulated as 0 cm (“near” condition, uncrossed, absolute disparity of nearest part: 0°) or 4.4 cm (“far” condition, uncrossed, absolute disparity of nearest part: 0.30°). Stimuli selected from a set of eight conditions (2 shapes × 2 orientations × 2 depth positions) were presented using a block design (Figure 1D). To avoid adaptation and maintain neuronal activation, the stimulus in each block was flashed on and off repeatedly for 0.5 s. Each time a stimulus pattern was shown, the random dots were regenerated. Each stimulus block comprised one of the eight conditions and lasted 15 s, and each stimulus condition was shown in two randomly selected blocks per run. After each stimulus block, the participant was required to make a judgment of the shape of the surface by pressing the corresponding button on a keypad. The judgment was followed by a 6 s fixation block. There was no fixation block following the final judgment block. Each run began with a 12 s fixation block. The total time taken for one run is calculated as follows: (1) the time of all stimulus blocks is 15 × 8 × 2 = 240 s; (2) the time of all blocks for judgment is 3 × 8 × 2 = 48 s; and (3) the time of fixation blocks is 12 + 6 × (8 × 2 − 1) = 102 s. Therefore, each run lasted a total of 240 + 48 + 102 = 390 s. Participants were required to observe the fixation marker and avoid any head movement during all runs. Any runs with excessive head movement were discarded. Excessive head movement was defined as head movement of >2 mm or head rotation of >2° during each run. Overall, participants were able to maintain their head static and at least six usable echo-planar imaging (EPI) scans were obtained for each participant.

### fMRI Data Acquisition

Imaging was performed using a 3 Tesla Siemens Verio MRI scanner with a 24-channel multi-phase array head coil at the Brain Communication Research Centre of the Kochi University of Technology. Participants’ heads were fixed with foam padding to reduce movement. For each participant, a high resolution T1-weighted anatomical scan (1 × 1 × 1 mm) was obtained to construct the exact inflated and flattened cortical surface. For the experimental scans, BOLD signals were measured using an EPI sequence [echo time (TE): 30 ms; repetition time (TR): 3,000 ms; number of volumes per run: 130; slice thickness: 3 mm; slice acquisition order: interleaved] from 35 slices covering the visual cortex, posterior parietal cortex, and posterior temporal cortex. In addition, a T2-weighted structural image was obtained in a run of 2.5 min and it was recorded at the same position as the slices of the corresponding EPI data in one session. Structural data were used as reference slices for 3D motion correction of EPI data as well as co-registration between T1-weighted anatomical images and EPI images. Following co-registration between anatomical and functional data in native anatomical space, all data were converted to Talairach coordinates.

**Figure 2:**
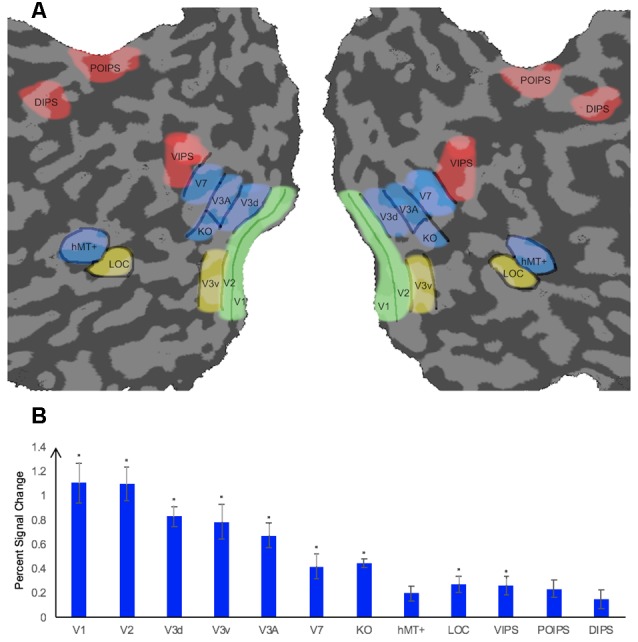
Illustration of a flattened cortical surface with regions of interest (ROIs)superimposed and percent signal change of each ROI. **(A)** ROIs. The retinotopic areas (V1, V2, V3d, V3v, V3A), higher ventral area (LOC), higher dorsal areas (KO, V7, hMT+), and intraparietal sulcus (IPS) areas [dorsal intraparietal sulcus (DIPS), parieto-occipital intraparietal sulcus (POIPS), ventral intraparietal sulcus (VIPS)]. Sulci are indicated in dark gray whereas gyri are indicated in light gray. ROIs were defined by standard localizers in separate sessions (see the “fMRI Data Acquisition” section). **(B)** Percent signal change of each ROI. Error bars represent standard error of the mean across participants (*n* = 8). Asterisks indicate that the percent signal change is significantly greater than 0, as assessed by a *t-test* of group data (*p ≤ 0.004).

We measured the ROIs (Figure 2A) for each participant using standard procedures in separate sessions prior to the main experiment. Retinotopically organized visual areas, namely V1, V2, V3d, V3v, and V3A, were localized by rotating wedge stimuli and expanding concentric rings (Sereno et al., 1995; DeYoe et al., 1996; Warnking et al., 2002). Area V7 was defined as the region anterior and dorsal to V3A with a lower visual field quadrant representation (Tootell et al., 1998; Tyler et al., 2005). Moreover, we identified some higher ventral areas including the LOC, the higher dorsal areas hMT+ and KO, and areas along the IPS including VIPS, POIPS, and DIPS using special independent localizers. LOC was identified as a set of continuous voxels in the lateral occipito-temporal cortex that showed significantly stronger activation (*p* < 10^−4^) to intact vs. scrambled images of objects (Kourtzi and Kanwisher, 2000, 2001). hMT+ was defined as a set of continuous voxels in the lateral temporal cortex that demonstrated significantly stronger activation (*p* < 10^−4^) to a set of coherent outward and inward moving dots compared to static dots (Zeki et al., 1991). KO was identified as a set of voxels that responded significantly stronger activation (*p* < 10^−4^) to motion-defined contours than the transparent motion of a field of black and white dots (Dupont et al., 1997; Zeki et al., 2003). Stimuli comprising nine randomly connected lines were used to locate the IPS areas (VIPS, POIPS, and DIPS). These areas were identified by contrasting activity to 3D shapes, which was produced by rotating stimuli in depth, vs. activity to 2D shapes, which was produced by moving stimuli along a frontoparallel plane (Vanduffel et al., 2002).

### Data Analysis

#### Pre-processing

Data processing and analysis were performed using the Freesurfer software package (Fischl, 2012), BrainVoyager QX (Version 2.8.4.2645, 64-bit; BrainInnovation, Maastricht, Netherlands), MATLAB R2014a (The MathWorks, Natick, MA, USA), and SPSS Statistics 23 (IBM Inc., Armonk, NY, USA). Freesurfer was used to remove the scalp of the T1-weighted 3D anatomical image per participant and segment the remaining parts into different regions, such as those of the outer skull and inner skull. The white matter (WM) was separated from the other components and later used as a mask to segment WM and gray matter (GM) in BrainVoyager QX. The brain was extracted from the skull and other tissues. Thereafter, we used BrainVoyager QX to transform the extracted brain into standard Talairach space. The flattened cortical surface of each participant was generated by segmenting the cortical surface along the GM/WM boundary, inflating the segmented GM, cutting along the calcarine sulcus, and flattening. The flattened cortical surface was used for visualizing functional maps and delineating ROIs, which were later used in the MVPA procedure. For the EPI data, 3D motion correction was performed (Woods et al., 1998) referencing the T2-weighted image obtained at the beginning of each session. No spatial smoothing was performed. Finally, co-registration was performed to align the functional EPI data to the T1-weighted anatomical data, and the functional EPI data were transformed into Talairach space.

#### ROI-Based Univariate Analysis

Univariate analysis was performed to assess whether the overall BOLD signal pattern in each selected ROI was higher for all stimuli compared with that at the fixation baseline. We calculated the “percent signal change” for the stimuli vs. the fixation baseline. The fMRI time courses were shifted by two volumes (6 s) to account for the hemodynamic delay of the BOLD signal. The percent signal change values were averaged across all runs and all participants.

SPSS Statistics 23 was used for statistical hypothesis testing. One-sample *t*-tests were used to assess whether percent signal change was significantly higher than chance level for each ROI.

#### ROI-Based MVPA

MVPA method is widely used to identify the global patterns of the cortical areas of the human brain and is useful for discovering function of the cortical areas. It allows the detection of subtle differences between the conditions of interest but it may bear unexpected pitfalls (Alizadeh et al., 2017). We carefully performed a series of MVPA for the EPI data for each ROI in MATLAB. SVM was used as the classifier for MVPA. The implementation of SVM was provided by MATLAB. Typically, two types of classifications were performed. The first was a same-type stimuli classification wherein we trained and tested SVM on the same type of data. This was performed to investigate whether specific patterns were contained in the dataset. The second was a transfer classification wherein we trained and tested SVM on different types of data. This was performed to assess whether a pattern contained in one type of data was also contained in another type of data. This is a useful method for determining whether a specific pattern is shared by different datasets.

Our MVPA procedure is as follows. Due to the hemodynamic delay of the BOLD signal, the fMRI time courses were shifted by two volumes (6 s). For each ROI in Talairach space, voxels were selected from both hemispheres and sorted from the largest to smallest according to their response (*t*-statistic) to the stimulus conditions compared with the fixation baseline. Using the *t*-statistics from the “stimulus vs. fixation baseline” contrast, the top 250 voxels were selected for all ROIs and all participants. For ROIs of participants for whom 250 voxels were unavailable, the highest number of voxels available was used for the classification.

To estimate the value for each voxel of the ROIs used as input for SVM for each stimulus block, the average value of the first three volumes after stimulus onset (*avg1*) and the average value of the last two volumes before stimulus onset (*avg2*) corresponding to the fixation baseline (after shifting by two volumes) were calculated. The *avg1 − avg2* difference was then calculated. Thereafter, the time series differences were transformed into *z*-scores and transmitted to SVM for training and testing.

The leave-one-run-out cross-classification method was used to evaluate the performance of the MVPA classification, i.e., data were partitioned according to the run they belong to, and data from one run were used for testing, whereas data from other runs were used for training. This procedure was repeated with different partitions of the data. The classification accuracy for each ROI was calculated as the average classification accuracy of all cross-classifications. The classification accuracies of all participants were averaged for each ROI.

To estimate the baseline for statistical significance, we performed classification analysis with randomly permutated fMRI patterns for all ROIs, i.e., we randomized the correspondences between fMRI data and labels and performed classification similar to that performed for normal non-permutated data. This procedure was repeated 1,000 times to create a distribution of classification accuracies. We used the 99.6th percentile (one-tailed, 12 ROIs) as the baseline for statistical significance.

## Results

### ROI-Based Univariate Analysis

The average percent signal changes across all runs and all participants per ROI are shown in Figure 2B. All ROIs showed a percent signal change >0 for the condition stimuli vs. fixation baseline, indicating that the BOLD signal from these ROIs were more strong during the stimuli condition than in the fixation baseline condition. However, the percent signal change was not significantly greater than 0 for the dorsal area hMT+ and parietal areas POIPS and DIPS. The reason for the absence of a significant change by these ROIs could be the trend in which signal changes increasingly became weaker from the early visual areas to higher visual areas, that the signal changes in these three areas were relatively weak, and that our criterion for significance was strict (*p ≤ 0.004). Regardless, they still showed an average signal change greater than 0.

### ROI-Based MVPA

#### Classification Accuracy for the Sign of Curvature Discrimination

**Figure 3:**
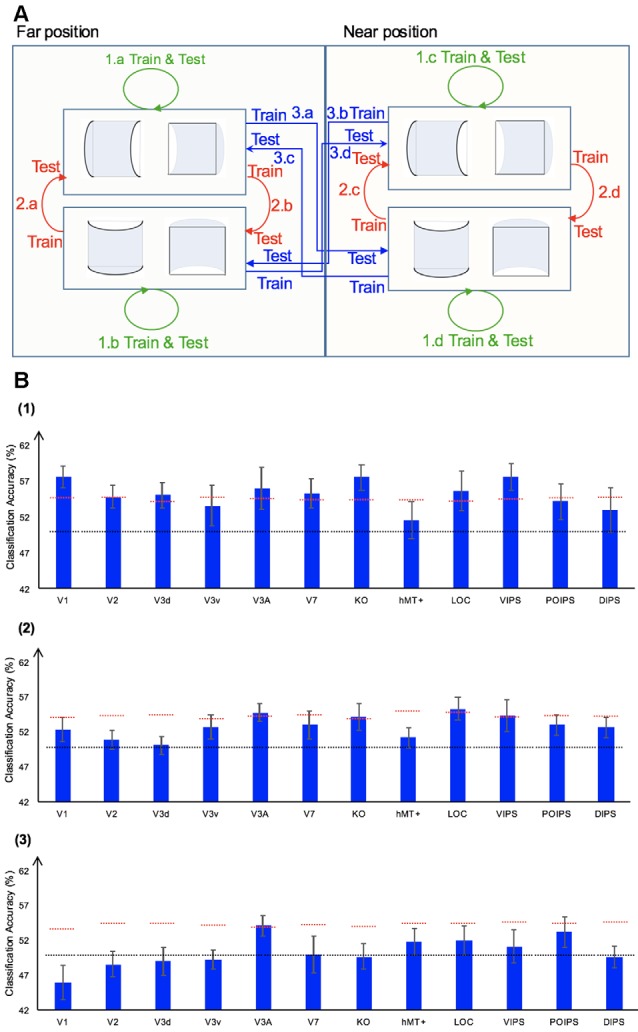
Sign of curvature classification and results. **(A)** Illustration of the types of the sign of curvature classification performed. 1.a, 1.b, 1.c, and 1.d represent same-type stimuli classification. 2.a, 2.b, 2.c, and 2.d represent transfer the sign of curvature classification of surfaces at the same depth position. 3.a, 3.b, 3.c, and 3.d represent transfer the sign of curvature classification of surfaces at different depth positions. **(B)** The accuracy of classification for convex vs. concave discrimination. (1) The accuracy of classification for the sign of curvature discrimination for same-type stimuli. (2) The transfer sign of curvature classification of surfaces at the same depth position. (3) The transfer sign of curvature classification of surfaces at different depth positions. The red horizontal dotted lines indicate the baselines of statistical significance for ROIs. The locations of these lines indicate the upper 99.6% percentile of the distribution of the accuracy of classification of the permutated data. The black horizontal line indicates the chance level for two-class classification. The error bars depict the standard error of the mean across subjects (*n* = 8).

We performed three types of the sign of curvature classifications (Figure 3A) as follows: (i) same-type stimuli sign of curvature classification (denoted by green arrows); (ii) transfer sign of curvature classification of the surfaces at the same depth position (denoted by red arrows); and (iii) transfer sign of curvature classification of the surfaces at different depth positions (denoted by blue arrows).

##### Same-Type Stimuli Sign of Curvature Classification

To investigate whether ROIs are selective to the sign of curvature of convex and concave surfaces, “same-type stimuli sign of curvature classification” was performed. Each stimulus exhibited the following three attributes: position (near or far), orientation (horizontal or vertical), and sign of curvature (plus or minus). Here, the “same-type stimuli sign of curvature classification” refers to trained and tested SVM using the same type of data, i.e., both the training and the testing data were of the same depth position (near or far) and orientation (horizontal or vertical). For example, SVM was trained on (horizontal, near) surfaces data and then tested using the same type of data (horizontal, near) surfaces. Similarly, we trained SVM on [(horizontal, far), (vertical, near), and (vertical, far)] surfaces separately and then tested SVM on the corresponding types of data (see 1.a, 1.b, 1.c, 1.d of Figure 3A). The average classification accuracy values across the four types of data of the eight participants are shown in Figure 3B–1.

All areas showed an average classification accuracy higher than chance level for the two-class classification (50%). The V1; V2; dorsal areas V3d, V3A, V7, and KO; ventral area LOC; and parietal area VIPS exhibited a classification accuracy higher than the baseline of statistical significance, suggesting that these areas are related to the processing of the sign of curvature of curved surfaces.

##### Transfer Sign of Curvature Classification of Surfaces at the Same Depth Position

The ability of SVM to classify the sign of surface curvature with accuracy higher than the baseline of statistical significance is attributable to two main reasons. One is that the BOLD signal patterns in ROIs reflect the local disparity of surfaces. The local disparity between surfaces with a different sign of curvature was different; it is possible that the multi-voxel BOLD signal patterns in ROIs reflect these differences and SVM used the differences for classification. The second possibility is that the multi-voxel BOLD signal pattern reflects a more generalized sign of surface curvature representation and SVM classified surfaces based on these patterns.

To assess whether each ROI is related to binocular disparity or more generalized processing of sign of curvature representation, the transfer sign of curvature classification of surfaces at the same depth position was performed.

There are three points that should be mentioned as follows: (1) the sign of surface curvature classification was performed by classifying whether a convex or concave surface was shown during the stimulus blocks; (2) the training and testing data were selected from blocks when stimuli were shown at the same depth condition; and (3) “transfer” indicates that the data used for training and testing had different attributes in terms of orientation (horizontal or vertical). For example, we trained SVM using data in which horizontal surfaces were shown and then tested SVM using data in which vertical surfaces were shown at the same depth position and vice versa. The classification is illustrated in 2.a, 2.b, 2.c, and 2.d of Figure 3A. The average accuracy values for the four classification types across the eight participants are shown in Figure 3B–2. In the higher dorsal areas V3A and KO, ventral area LOC, and parietal area VIPS, the fMRI responses evoked by one type of surfaces (horizontal or vertical) could allow the sign of curvature classification of responses evoked by the other type of surfaces (vertical or horizontal, respectively). Because the disparity patterns of the surfaces used for training and testing were different, this result was probably related to more generalized processing of the sign of surface curvature.

To further investigate whether generalized representation is invariant of depth position, we performed the transfer sign of curvature classification of surfaces at different depth positions.

##### Transfer Sign of Curvature Classification of Surfaces at Different Depth Positions

In this type of classification, SVM was trained to classify the sign of curvature of surfaces. “Transfer” indicates that the data used for training and testing have different attributes in terms of both depth position and orientation. For example, we trained SVM using data with horizontal surfaces in the far position and tested SVM using data with vertical surfaces in the near position. These classifications are illustrated by arrows 3.a, 3.b, 3.c, and 3.d of Figure 3A. The average accuracies for the four types of classification across eight participants are shown in Figure 3B–3.

As shown, only the classification accuracy for V3A was higher than the baseline of statistical significance (classification accuracy: 54.05%; baseline of statistical significance: 53.92%). This indicates that V3A is related to a more generalized representation of sign of surface curvature and is invariant to depth position.

**Figure 4:**
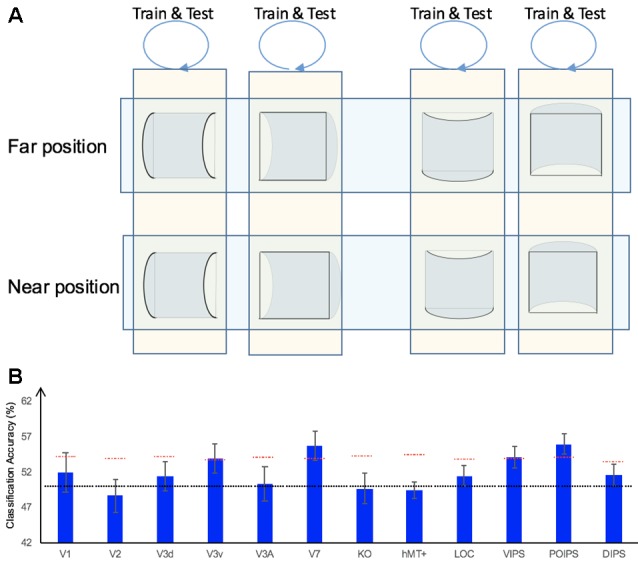
Near–far classification and results. **(A)** Illustration of the near–far classification. We trained and tested support vector machine (SVM) using the same type of data. **(B)** The accuracy of classification for near vs. far discrimination using same-type stimuli. The red horizontal dotted lines indicate the baselines of statistical significance for ROIs. The locations of these lines indicate the upper 99.6% percentile of the distribution of the accuracy of classification of the permutated data. The black horizontal line indicates the chance level for two-class classification.

Alternatively, the near–far information between the center and surrounding of the surfaces could support all the three types of sign of surface curvature classification. Moreover, for “the same-type stimuli sign of curvature classification” and “transfer sign of curvature classification of surfaces at the same depth position,” the different depth positions of peaks of surfaces with a different sign of curvature could be used for classification. To exclude this possibility, we investigated the classification accuracy for near vs. far surface discrimination. Figure 4A illustrates the near–far classification performed. We performed same-type stimuli near–far classification. SVM was trained to classify a near vs. far position of the surfaces. “Same-type stimuli” indicates that the data used for training and testing exhibited the same sign of curvature (plus or minus) and same orientation (horizontal or vertical). For example, we trained and tested SVM using data with horizontal surfaces with minus curvature. The average classification accuracies for the four types of data across all participants are shown in Figure 4B.

As shown, the average classification accuracy for V3A was approximately that of chance level for two-class classification, indicating that near–far surfaces cannot be classified by V3A, at least in our experimental setting. By combining our findings thus far, we can conclude that the higher-than-baseline accuracy for the sign of curvature classification in V3A was not likely to be based on the multi-voxel BOLD signal pattern that reflect near–far information between the center and side of the stimulus (because we cannot classify near–far surfaces by V3A in our experimental setting); rather, it is likely related to the pattern that reflects the generalized sign of surface curvature representation. This result is consistent with our hypothesis that the dorsal area V3A is a candidate involved in generalized sign of surface curvature representation.

The results showed high accuracy in the three types of the sign of curvature discrimination in V3A. The information used for the discrimination, however, may be different: (1) for the “same-type stimuli sign of curvature classification,” it may be explained by local disparities (or zero-order disparity) because of the training data and testing data were of identical type; (2) for the “transfer sign of curvature classification of surfaces at the same depth position,” it is may be classified according to the different depth positions of the peaks of surfaces with the different sign of curvature or near–far information between the center and surroundings of the surfaces. However, we also checked “same-type stimuli near–far classification,” for which disparities were different between near and far surfaces. The result showed that in our experimental condition, the accuracy of the classification was approximately that of chance level in V3A. Considering this finding, we can infer that it was unlikely to be classified by these two reasons; and (3) for the “transfer sign of curvature classification of surfaces at different depth position,” the disparity of surfaces for training (e.g., horizontal surfaces in the near depth position) and disparity of the surfaces for testing (e.g., vertical surfaces in the far depth position) were quite different. Hence, it is unlikely to be classified by local disparities. In addition, V3A is on the dorsal visual stream, and this visual stream is related to coarse stereopsis. It is likely that V3A was involved in the representation of the sign of surface curvature in a more abstract (coarse) manner, which did not depend on the orientation of the surface and depth position. Therefore, V3A showed high accuracy in this classification. Notably, the high accuracy demonstrated by V3A showed in our classification does not indicate that the ventral stream areas were not involved in the generalized representation of shape. There is an exchange of information between dorsal and ventral streams (Yeatman et al., 2014; Sim et al., 2015; Takemura et al., 2016). We observed that V3A showed statistical significance in all the three types of sign of curvature classification. In conclusion, V3A is a crucial part of the process related to the more generalized sign of surface curvature representation, and the results are consistent with those reported in previous studies.

#### Classification Accuracy for Horizontal vs. Vertical Surfaces Discrimination

**Figure 5:**
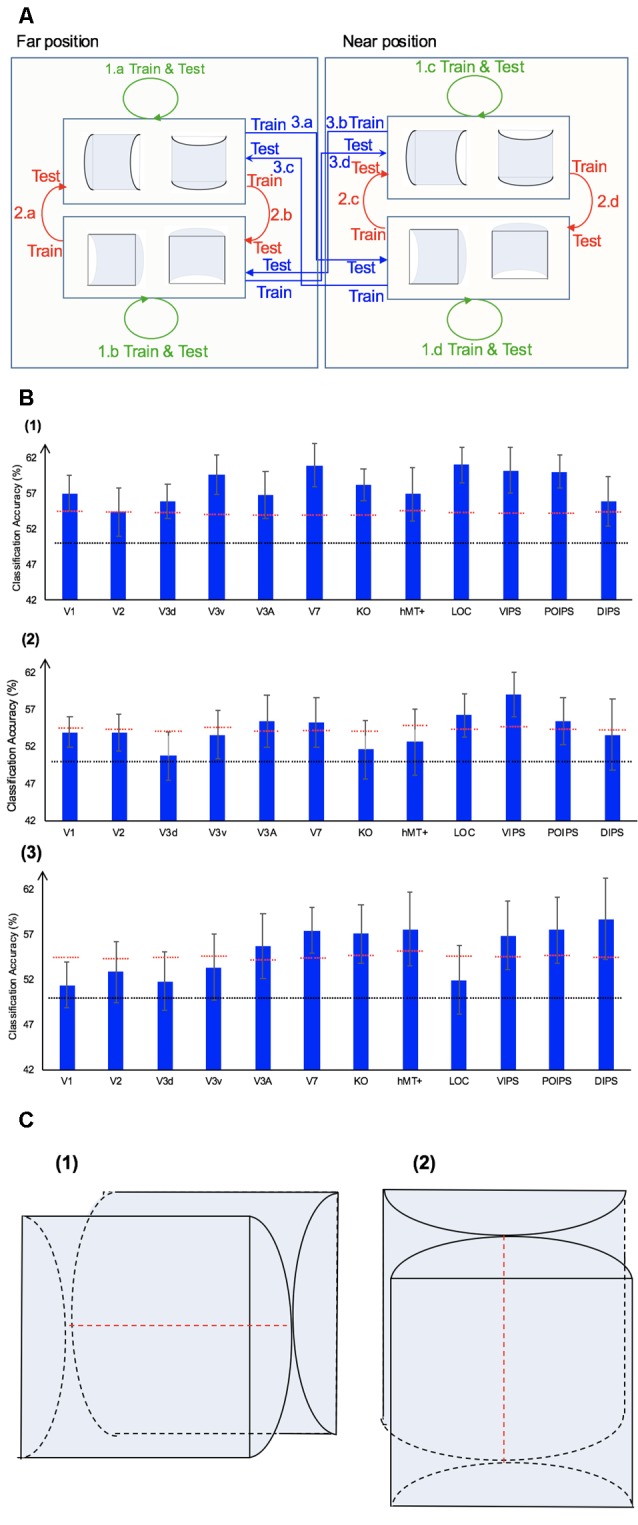
The horizontal–vertical classification, results and local disparity for classification. **(A)** Illustration of the horizontal–vertical classification. 1.a, 1.b, 1.c, and 1.d indicate same-type stimuli horizontal–vertical surfaces classification. 2.a, 2.b, 2.c, and 2.d indicate transfer horizontal–vertical classification of surfaces with different signs of curvature at the same depth position. 3.a, 3.b, 3.c, and 3.d indicate transfer horizontal–vertical classification of surfaces with different signs of curvature at different depth positions. **(B)** The accuracy of classification for horizontal vs. vertical discrimination: (1) The accuracy of classification for same-type stimuli horizontal–vertical surfaces classification. (2) The accuracy of classification for transfer horizontal–vertical classification of surfaces with different signs of curvature at the same depth position. (3) The accuracy of classification for transfer horizontal–vertical classification of surfaces with different signs of curvature at different depth positions. The red horizontal dotted lines indicate the baselines of statistical significance for ROIs. The locations of these lines indicate the upper 99.6% percentile of the distribution of the accuracy of classification of the permutated data. The black horizontal line indicates the chance level for two-class classification. **(C)** A schematic illustration of two surfaces of different shapes of the same orientation at different depth positions that share some similar disparity patterns. (1) A horizontal concave surface in the near position and a horizontal convex surface in the far position are shown. These two surfaces share a similar disparity pattern around the position shown by the horizontal red dotted line. (2) Vertical concave surface in the near position and vertical convex surface in the far position. These two surfaces share a similar disparity pattern around the vertical dotted red line.

In addition to the representation of the sign of surface curvature defined by disparity, we investigated the representation of orientation of stereoscopic surfaces among ROIs. Accordingly, ROI-based MVPA was performed. The following three types of horizontal vs. vertical surface classifications were conducted (Figure 5): (i) the same-type stimuli horizontal–vertical surfaces classification; (ii) the transfer horizontal–vertical classification of surfaces with different signs of curvature at the same depth position; and (iii) the transfer horizontal–vertical classification of surfaces with different signs of curvature at different depth positions.

##### Same-Type Stimuli Horizontal–Vertical Surfaces Classification

We trained SVM to classify the orientation of surfaces, i.e., to judge whether surfaces were horizontal or vertical. “Same-type stimuli” indicate that both the training and testing data were of the same sign of curvature (plus or minus) and depth position (near or far). For example, we trained and tested SVM using data with horizontal–vertical surfaces with minus sign of curvature in the near position. This classification is illustrated in 1.a, 1.b, 1.c, and 1.d of Figure 5. The average classification accuracies for all data types across the eight participants are shown in Figure 5B–1.

The accuracies of classification for distinguishing horizontal vs. vertical orientation among all areas were higher than the baseline of statistical significance, indicating that multi-voxel pattern in all ROIs contains robust orientation information.

##### Transfer Horizontal–Vertical Classification of Surfaces With Different Sign of Curvature

This classification included two types as follows: the “transfer horizontal–vertical classification of surfaces with different signs of curvature at the same depth position” and the “transfer horizontal–vertical classification of surfaces with different signs of curvature at different depth positions.”

The reasons for SVM showing a horizontal–vertical classification accuracy higher than the baseline of statistical significance for same-type stimuli horizontal–vertical surfaces may be the following. First, the disparity patterns of horizontal surfaces and vertical surfaces were different. The multi-voxel patterns could reflect this disparity, and the SVM used the differences for the classification. Second, the multi-voxel patterns could reflect horizontal and vertical orientation representation processing, i.e., these multi-voxel patterns reflect the representation of horizontal–vertical orientations of surfaces and SVM classified the surfaces based on these patterns. To investigate whether the significantly high accuracy for each ROI depended on disparity or more generalized horizontal–vertical orientation representation irrespective of sign of surface curvature, “transfer horizontal–vertical classification of surfaces with different sign of curvature at the same depth position” was performed. SVM was trained to classify whether a stimulus shown to a participant was horizontal or vertical. “Transfer” indicates that the stimuli used for training and testing exhibited different sign of curvature but the same depth position. For example, data of surfaces with a minus sign of curvature shown in the near position were used to train SVM and data of surfaces with a plus sign of curvature shown in the near position were used to test SVM. The classification is illustrated in 2.a, 2.b, 2.c, and 2.d of Figure 5A. The average accuracies of classification for the four types of classification across all participants are shown in Figure 5B–2. In the dorsal areas V3A and V7, higher ventral area LOC, and parietal areas VIPS and POIPS, the fMRI responses evoked by one type (minus or plus) of horizontal–vertical surface can allow the classification of the response evoked by another type (plus or minus, respectively) of horizontal–vertical surface at the same depth position. This finding suggests that these areas are related to the generalized processing of orientation of stereoscopic surfaces irrespective of disparity types.

For the “transfer horizontal–vertical classification of surfaces with different signs of curvature at different depth positions,” SVM was trained to classify the horizontal–vertical orientation of the surface of a stimulus shown to participants. “Transfer” indicates that the data used for training and testing had different attributes in sign of curvature (plus or minus) and depth (near or far). For example, SVM was trained using data with a surface with a minus sign of curvature in the near position and SVM was tested using data with a surface with a plus sign of curvature in the far position. These classifications are shown by arrows 3.a, 3.b, 3.c, and 3.d in Figure 5A. However, there is other information that could support this type of horizontal–vertical classification, namely there are some common disparity patterns between the surfaces of different sign of curvature and the same orientation at different depth positions. In detail: (1) for the pair [(horizontal, concave, near) surface and (horizontal, convex, far) surface] and the pair [(vertical, concave, near) surface and (vertical, convex, far) surface], the former pair of surfaces share a common disparity pattern at the horizontal red line shown in (1) in Figure 5C; the latter pair of surfaces share a common disparity pattern at the vertical red line in (2) in Figure 5C. The difference between the former and latter common patterns can be used for horizontal–vertical classification, wherein an SVM was trained on concave surfaces in the near position and tested on convex surfaces in the far position and vice versa (3.c and 3.a in Figure 5A); and (2) for the pair [(horizontal, convex, near) surface and (horizontal, concave, far) surface] and pair: [(vertical, convex, near) surface and (vertical, concave, far) surface], the former pair of surfaces share a common disparity pattern at the uppermost and lowermost horizontal lines; the latter pair of surfaces share a common disparity pattern at the most left and right vertical lines. The difference between the former and latter common patterns can be used for horizontal–vertical classification, wherein an SVM was trained on a convex surface in the near position and tested on a concave surface in the far position and vice versa (3.b and 3.d in Figure 5A). Therefore, whether the representation is invariant of depth position cannot be verified. The average accuracies of all classifications across the eight participants are shown in Figure 5B–3. As shown, in addition to the areas (except the LOC) with an accuracy of classification higher than the baseline of statistical significance for “transfer horizontal–vertical classification of with different sign of curvature at the same depth position,” those of the dorsal areas KO and hMT+ and the parietal area DIPS are higher than the baseline of statistical significance. For the LOC, accuracy is higher than the baseline of statistical significance in “transfer horizontal–vertical classification of surfaces with different signs of curvature at the same depth position” but not for “transfer horizontal–vertical classification of surfaces with different signs of curvature at different depth positions.” This is possible because the LOC is selective to depth position. Preston et al. (2008) showed that the LO, a sub-region of the LOC, contains information regarding the depth position of planes depicted by an RDS.

In summary, although we were unable to confirm that the orientation representation is invariant with depth position, we can conclude that the dorsal areas V3A and V7, the higher ventral area LOC, and the parietal areas VIPS and POIPS are somewhat related to the generalized representation of horizontal–vertical orientation of surfaces. In addition, because in the LOC, accuracy in “transfer horizontal–vertical classification of surfaces with different signs of curvature at different depth positions” was lower than the baseline for statistical significance, we can confirm that the orientation representation in the LOC is not invariant with depth position.

## Discussion

In the present study, we used the MVPA method and fMRI data to investigate the sign of curvature and orientation representations of 3D surfaces defined by binocular disparity. By comparing a series of the accuracies of classification across ROIs, we found that all types of sign of curvature classification—including the “same-type stimuli sign of curvature classification,” “transfer sign of curvature classification of surfaces at same depth position,” and “transfer sign of curvature classification of surfaces at different depth positions”—showed an accuracy of classification higher than the baseline of statistical significance in the dorsal area V3A. For horizontal–vertical orientation classification, the accuracies of classification for both “same-type stimuli horizontal–vertical classification” and “transfer horizontal–vertical classification of surfaces with different signs of curvature at same depth position” were significant in the dorsal areas V3A and V7, higher ventral area LOC, and parietal areas VIPS and POIPS. In summary, these results indicate that the dorsal area V3A is related to the generalized representation of the sign of curvature and that some dorsal and ventral areas, as well as parts of IPS, are related to the generalized horizontal–vertical orientation representation of surfaces defined by disparity.

### Comparison With Earlier Studies of Shape Representation

In the “two-stream” theory of cortical areas, visual information is processed progressively from the early visual areas to higher areas and divided into two anatomically and functionally separate streams after V1. Each stream processes visual information in a hierarchical manner, with each area processing information based on the results of previous areas (Zeki, 1978). Mishkin and Ungerleider (1982) first conceptualized this theory based on lesion research of non-human primates. The ventral stream has been termed the “what” stream whereas the dorsal stream has been termed the “where” stream, as the ventral stream is related to an object’s shape and identity whereas the dorsal stream is related to object’s location and spatial relationships. According to this perspective, 3D shape representation should exist on the ventral stream. More recently, a revised “two-stream” theory was offered by Goodale and Milner (1992). Rather than viewing both streams as contributing to conscious visual awareness, they argue that only the ventral stream contributes to conscious vision (known as the “perception” stream). Information in the dorsal pathway is used for the unconscious control of action, such as the movement of the body guided by visual input (known as the “action” stream). From this view, shape representation can exist on both the ventral and dorsal pathways with representation in the ventral stream serving as perception and in the dorsal stream as visually guided action. There is ample evidence that 3D shape information from monocular cues is processed both in the ventral and dorsal visual streams in humans (Orban et al., 1999; Paradis et al., 2000; Taira et al., 2001; Georgieva et al., 2008). In macaques, stereo information has been found in both the dorsal and ventral visual stream (Parker, 2007). Further, in an fMRI study of patients suffering from visual object agnosia due to a ventral cortex lesion (dorsal cortex is intact), Freud et al. (2017) found that the dorsal cortex can mediate object representations that are dissociable from object representations in the ventral stream, and together with other evidence, they claimed that representations in the dorsal stream mediate the processing of object-related structural information, but are insufficient for normal object perception. However, there is no complete agreement on the distinction between the two pathways and the role played by each area on the pathways. Ventral “perception” and dorsal “action” separation has been challenged by findings that dorsal areas are also involved in shape representation for tasks that do not involve visually guided action (Sereno et al., 2002; Lehky and Sereno, 2007; Konen and Kastner, 2008).

Specifically, for the representation of shape from disparity, disparity-selective neurons are widely distributed throughout the visual cortical areas, from as early as the striate cortex (V1 or primary visual cortex) to extra-striate and higher areas like the posterior parietal and inferior temporal cortices in both the dorsal stream and ventral visual streams (Neri et al., 2004). In the early visual area V1, neurons show selectivity for absolute disparity of RDS stimuli (Cumming and Parker, 1997). The neurons in V2 show selectivity for absolute and relative disparities (Thomas et al., 2002) and can transform absolute disparity to relative disparity during 3D vision, which is important for invariant object recognition (Grossberg et al., 2011). Besides the V3A, the dorsal regions, including V3B/KO and V7, are involved in processing disparity-defined depth (Preston et al., 2009). The neurons’ selectivity for binocular disparity creates a foundation for ROIs showing accuracy for classifying stereoscopic shapes above the baseline of statistical significance in our experiment, whereas the wide distribution of these neurons suggests that several areas, from the early visual areas to higher areas, offer a high-level accuracy of classification.

In the present study, we first examined the same-type stimuli sign of curvature classification. The ROIs are involved in processing of the sign of surface curvature were found in both the dorsal (V1, V2, V3d, V3A, V7, KO, and VIPS) and ventral (V1, V2, and LOC) streams. This finding is consistent with the two-stream theory, in which shape representation in the ventral stream mediates shape perception, while shape representation in the dorsal stream mediates visually guided actions. However, we cannot be certain whether the sign of curvature representations in the dorsal areas are involved in visually guided actions or not, although our results support that this representation can exist in the dorsal areas. Therefore, this question requires further research.

Besides investigating the involvement of processing the sign of curvature of surfaces defined by disparity in ROIs, we investigated whether the ROIs are involved in the processing of low-level binocular disparity or more a generalized representation, which may relate to perception or visually guided actions. This was performed by the “transfer sign of curvature classification of surfaces at the same depth position” and “transfer sign of curvature classification of surfaces at different depth positions” analyses; the analyses revealed that all classification accuracies in the dorsal area V3A were higher than the baseline of statistical significance, indicating that V3A is involved in the more generalized representation of sign of surface curvature that does not depend on local disparity pattern (orientation and depth position).

To the best of our knowledge, there is no direct research on the generalized representation of the sign of curvature of surfaces solely defined by disparity. However, there is some related research. Dövencioğlu et al. (2013) adopted convex “bumps” and concave “dimples” defined by shading, binocular disparity, and their combination. By comparing pattern classifications in different visual cortical areas, they found that fMRI BOLD signals in the dorsal visual area V3B/KO were more discriminable when disparity and shading concurrently signaled depth, indicating the integration of these two cues. In a cross-cue transfer test, they found that the fMRI BOLD signal evoked by one type of cue could support the classification of a signal evoked by another type of cue, which indicates the generalized representation of shapes in the dorsal visual cortex by combining qualitatively different information with 3D perception. However, the results of the present study differ from those of Dövencioğlu et al. (2013). There are several possible reasons for this difference. First, we focused on 3D shape from binocular disparity whereas Dövencioğlu et al. (2013) focused on 3D shape from binocular disparity and shading. Our research revealed a common multi-voxel BOLD signal patterns caused by the surface of same shape from different disparity patterns, whereas Dövencioğlu et al.’s (2013) research revealed a common multi-voxel BOLD signal pattern caused by the same shape from different cues. Second, the convex–concave shapes used in our experiment and those in Dövencioğlu et al.’s (2013) experiment were different. We adopted horizontal-positioned and vertical-positioned hemi-cylindrical convex–concave surfaces, while they used convex “bumps” and concave “dimples” of hemispheric shapes. Nevertheless, our finding that V3A is involved in a more generalized shape representation does not contradict that of previous research: in macaques, clusters of disparity-selective neurons have been found in V3A (Anzai et al., 2011; Hubel et al., 2015); using a 7T fMRI scanner, Goncalves et al. (2015) showed that disparity-selective neurons in the visual cortical areas were clustered and that this organization persisted across imaging sessions, particularly in the V3A. These studies showed that V3A is disparity-selective and is possibly a locus that is related to a generalized representation of sign of surface curvature.

### Comparison With Earlier Studies on Orientation Representation

The perception of orientation is essential for reconstructing the 3D structure of an object. Previous studies have demonstrated that the parietal lobe plays an important role in orientation representation. Cues that support the depth discrimination of orientation include linear perspective and texture, velocity, and disparity gradients (Johnston and Passmore, 1994). These cues can be divided into the two following categories: monocular cues and binocular cues. In a study of alert monkeys, Shikata et al. (1996) found that neurons in the lateral bank of the caudal part of the intraparietal sulcus (CIP) were sensitive to the orientation of a surface defined by disparity. Neurons from this region were also shown to be selective to binocular disparity gradients in monkeys (Sakata et al., 1998; Taira et al., 2000). Tuning to disparity-defined 3D surface orientation has also been found in TE regions (Janssen et al., 2000).

In our study of orientation representation of 3D surfaces defined by disparity, we first investigated the whether ROIs are involved in orientation processing using the “same-type stimuli horizontal–vertical surfaces classification.” The accuracies of classification were higher than the baseline of statistical significance for all ROIs including the V1, V2, the ventral visual areas V3v and LOC, the dorsal visual areas V3d, V3A, KO, hMT+, and V7, and the IPS areas VIPS, POIPS, and DIPS. This finding is consistent with those of previous reports that disparity-selective neurons are widely distributed across visual cortical areas. To investigate the more generalized representation of horizontal–vertical orientation, we performed a “transfer horizontal–vertical classification of surfaces with different signs of curvature at the same depth position.” The results showed that from higher dorsal areas to the parietal areas V3A, V7, VIPS, and POIPS and ventral area LOC, the accuracies of classification were higher than chance level, suggesting that these areas are involved in the more generalized representations of orientation. The neurons in the CIP were previously shown to be sensitive to monocular texture gradient cues as well as to disparity, suggesting that they integrate texture and disparity gradient signals to construct a generalized representation of 3D surface orientation (Tsutsui et al., 2002). Ban and Welchman (2015) inferred the computational hierarchy that supports the estimations of slant using fMRI measurements and a series of generative models, and found that V3A was involved in slanted surface information representation by pooling disparity across space; the representation was largely unaffected by low-level stimulus changes, and V3A response showed a degree of tolerance across different depth positions. They concluded that V3A, which anatomically precedes CIP in macaques, is an earlier locus for processing the disparity signals of slant. This finding is consistent with our finding that the accuracy of classification in V3A was higher than the baseline of statistical significance. In addition, we found that the areas V7, VIPS, and POIPS may be related to the generalized representation of orientation. This is consistent with previous animal research showing CIP involvement in orientation representation through monocular cues such as texture gradient and perspective as well as binocular cues of binocular disparity, suggesting that representation in this area may be more generalized (Tsutsui et al., 2001, 2002). Moreover, the relationship between human and monkey neuroanatomy is not exact; the human V7 and/or VIPS are considered to correspond to the macaque CIP (Orban et al., 2006a). Therefore, it is reasonable that the human areas V7 and VIPS were selective to orientation.

### Other MVPA Analysis

Janssen et al. (2001) showed that neurons in the TEs represent differences in the second-order disparities of 3D shapes. Moreover, they found that the neurons in TEs can code the orientation of curvature (i.e., vertical or horizontal). Therefore, one may believe that the representation of vertical gradients (as in horizontally oriented cylinders) and that of horizontal gradients (as in vertically oriented cylinders) should be different. However, this may not necessarily be true. In the aforementioned study, the “selectivity for horizontal compared with vertical 3D shapes” was investigated, and the following combinations were verified: (1) the neurons selective for vertical but not for horizontal 3D shapes; (2) neurons selective for both directions of curvature; and (3) neurons selective for horizontal but not for vertical. A total of 104 neurons were tested, and the result showed that 16 of those neurons were selective for both vertical and horizontal 3D shape. In this regard, there may be neurons that are selective for both types of surfaces in some areas of the visual cortex. For a comprehensive investigation, we examined two additional types of classification in which horizontal surfaces and vertical surfaces were independently analyzed.

**Figure 6:**
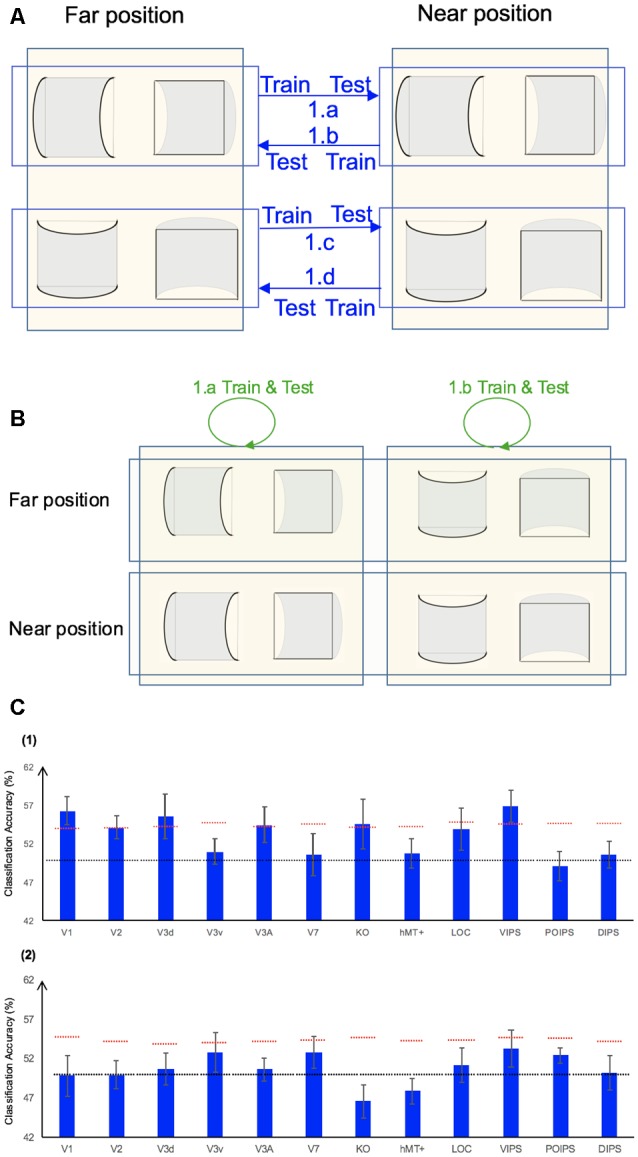
Additional analysis and results. **(A)** Illustration of the additional types of the sign of curvature classification performed. 1.a, 1.b, 1.c, and 1.d represent the transfer sign of curvature classification that trained the SVM using data of surfaces at the near position and tested the SVM using data of surfaces at the far position, and vice versa (denoted by A1). **(B)** Illustration of the additional near–far classification performed. We trained and tested the SVM using data of both convex and concave surfaces with the same orientation (denoted by A2). **(C)** Accuracy of the additional types of classification. (1) The accuracy of classification for the additional classification A1. (2) The accuracy of classification for the additional classification A2. The red horizontal dotted lines indicate the baselines of statistical significance for the ROIs. The locations of these lines indicate the upper 99.6% percentile of the distribution of the accuracy of classification of the permutated data. The black horizontal line indicates the chance level for two-class classification.

Two additional types of classification were also performed in our analysis. The first type was related to the sign of surface curvature classification, whereas the second type was related to the classification of the surface position in terms of depth. First, to investigate whether ROIs were involved in the generalized representation of the sign of curvature irrespective of the depth position of the surface, additional transfer sign of curvature classification was performed (hereafter denoted by A1). This classification is illustrated in Figure 6A (1.a, 1.b, 1.c, and 1.d). We trained the SVM using data of surfaces at the near position and tested using data of surfaces in the same orientation at the far position and vice versa. The average accuracy of the four types of classification across eight participants is shown in Figure 6C–1. The results showed that V1; V2; dorsal areas V3d, V3A and KO; and the parietal area VIPS exhibited an accuracy of classification that was statistically significant. Second, to identify the areas containing information regarding the depth position of the surface, an additional near–far classification was performed (hereafter denoted by A2). As illustrated in Figure 6B (1.a and 1.b), we trained and tested the SVM using data of surfaces for both the plus and minus sign of curvature in the same orientation. The average accuracy of the two types of classification across eight participants is shown in Figure 6C–2. The results showed that accuracy of classification in all ROIs did not reach the baseline of statistical significance.

Furthermore, the additional analysis showed the following:

1. For classification A1, the results were similar to those reported for “same-type stimuli sign of curvature classification” in “Same-Type Stimuli Sign of Curvature Classification” section (hereafter, this type of classification is denoted by S1), in which both the training and testing data were of the same depth position (i.e., near or far) and orientation (i.e., horizontal or vertical). However, the accuracy of classification in V7 and LOC was not significant. This observation can possibly be attributed to the representation of the sign of curvature V7 and LOC being more sensitive to the depth position. For the V7 area, this can be inferred from the results of the “same-type stimuli near–far classification” (Figure 4B). According to the results, V7, VIPS and POIPS showed a significant near–far accuracy of classification, whereas other areas did not. Therefore, the V7 did not show a significant accuracy in this additional classification A1. For the LOC area, although the accuracy of classification was not significant in near–far discrimination, Preston et al. (2008) showed LO (which is a sub-region of LOC) contained information regarding the depth position of planes depicted using a random dot stereogram. This may explain the lack of statistically significant accuracy for LOC area in this additional classification A1. The VIPS showed a significant accuracy in both classification A1 and near–far classification. This is probably because the VIPS contains a subgroup of neurons that are sensitive to the near–far depth position as well as neurons that are selective to the sign of curvature irrespective of the depth position.
2. For classification A2, the results were inconsistent with those previously reported. A study showed that numerous areas from the early visual cortex areas to higher areas contained near–far information regarding the stereoscopic plain. Preston et al. (2008) found that numerous areas, such as the retinotopic areas V1, V2, V3v and V4; higher ventral area LO; higher dorsal area hMT+; and IPS areas VIPS, POIPS, and DIPS showed a significant accuracy of classification. The discrepancy between the results of the present study, which showed no significant accuracy in all ROIs, and those reported by the previous study may be attributed to the following reasons. First, the stimuli used in the studies were different. We adopted curved surfaces in different depth positions, whereas Preston et al. (2008) adopted frontoparallel flat planes in different depth positions. It may be more difficult for the SVM to perform a near–far classification on curved than plain surfaces as the local disparity information is different within the curved surface. Second, we trained and tested the SVM using data of surfaces with a different sign of curvature. These surfaces with a different sign of curvature exhibited differences in local disparity, even at identical depth positions. Based on these reasons, we did not identify an ROI with a significant accuracy of classification.

### Limitations

In our experiment, we adopted curved surfaces with different signs of curvature and orientations. These surfaces were simulated in two different depth positions behind the fixation marker. For the surfaces in the same depth position, the surfaces with the same sign of curvature (i.e., minus or plus) and different orientations (i.e., horizontal or vertical) shared a similar average absolute disparity. In contrast, surfaces with a different sign of curvature (irrespective of orientation) at the same depth position exhibited differences in the average absolute disparity. For the surfaces in the different depth positions, the surfaces (irrespective of the sign of curvature) exhibited differences in the average absolute disparity.

For the sign of curvature classification, there were some limitations in our study: in the “transfer sign of curvature classification of surfaces at the same depth position,” we sought to investigate the generalized representation of the sign of surface curvature, irrespective of the orientation of surfaces. The differences in the average absolute disparity between surfaces with a different sign of curvature in the same depth position may have been used for this kind of sign of curvature classification. From this perspective, this may affect the conclusion of generalized representation of the sign of curvature (shape) of surfaces. However, most importantly, for the “transfer sign of curvature classification at different depth positions,” it was not possible to use the differences in average absolute disparity between surfaces with a different sign of curvature for classification. For this type of classification, the observed significantly high accuracy in the V3A cannot be interpreted as selectivity to average absolute disparity. However, it may only be interpreted as a more generalized sign of curvature representation of surfaces. Therefore, it is reasonable to conclude that V3A was involved in the more generalized representation of the sign of curvature of surface, irrespective of its orientation and depth position.

For the orientation classification, no problems were caused due to the mismatch in the absolute disparity between surfaces with a different sign of curvature. In our study, we mainly performed two types of horizontal–vertical classification, namely the “same-type stimuli horizontal–vertical surfaces classification” and “transfer horizontal–vertical classification of surfaces with different signs of curvature.” The significantly high accuracy for these two types of classification could not have been caused by the mismatch in the absolute disparity between the convex and concave surfaces. However, we identified a few areas showing significant classification accuracy, indicating the involvement of a more generalized orientation representation.

In this investigation, we only selected convex and concave surfaces in two different orientations. The reasons for the selection of these surfaces are discussed below.

First, the main focus of our study was to investigate the sign of curvature and the horizontal–vertical representation of the stereoscopic surface in ROIs and to assess the involvement of generalized representation in each ROI. For the investigation of generalized representation of the sign of curvature and orientation, we required stimuli that exhibit both a sign of curvature and orientation features. Moreover, the sign of curvature and orientation features can be easily and independently manipulated. Therefore, the convex and concave surfaces with two different orientations were considered. Other stimuli, such as “convex ‘bumps’ vs. concave ‘dimples”’ used by Dövencioğlu et al. (2013) only have the sign of curvature feature. Thus, they cannot be used to investigate the generalized sign of curvature representation or orientation representation.

Second, the surfaces we selected can be regarded as the element of a more complex surface (e.g., sinusoidal surface). We intended to initially investigate a simple and basic surface in our study first. Therefore, we selected convex and concave surfaces in two different orientations as our stimuli. We did not investigate a more complex index of surface, such as the Koenderink shape index (Koenderink and Doorn, 1992), because it is more complicated, and it may be hard to show surfaces with differences large enough (e.g., two surfaces with a large curvature difference), which is not suitable for the MVPA method.

Another concern in this study is that the number of participants is relatively small compared with some previous MVPA studies. In the study by Murphy et al. (2013), data from 12 participants were used for analysis. However, there are studies that have included fewer or the same number of participants than ours. In a study by Horikawa and Kamitani (2017), five participants were recruited, whereas in the study by Preston et al. (2008), eight participants were recruited in the experiment. In our study, we assured reliability by performing “permutation tests.” Permutation tests are widely used for significance testing in classification-based fMRI analyses (Ban and Welchman, 2015; Patten and Welchman, 2015). In our case, we randomized the correspondences between fMRI data and labels, and performed classification similar to that performed for normal, non-permutated data. This procedure was repeated 1,000 times to create a distribution of classification accuracies. We used the 99.6th percentile (one-tailed, 12 ROIs) as the baseline for statistical significance. This indicates that if a classification is higher than the baseline for statistical significance, it is extremely rare that the accuracy was obtained by chance in the eight participants. Therefore, we believe that our results are valid and reliable.

## Conclusion

In the present study, we first investigated whether ROIs are involved in the generalized sign of curvature representation of curved surfaces defined by binocular disparity. Moreover, the generalized horizontal–vertical orientation representation of these surfaces was also investigated. fMRI experiments using MVPA were conducted. Two types of classifications were performed as follows: (1) same-type stimuli classification, which was used to validate the selectivity to the sign of curvature or horizontal–vertical orientation in each ROI; and (2) transfer classification, which was used to validate the generalized representation.

Areas V1, V2, dorsal areas (V3d, V3A, V7, and KO), the ventral area (LOC), and the parietal area (VIPS) were found to be related to the sign of curvature. Among these areas, V3A showed a more generalized sign of curvature representation irrespective of surface orientation and depth position.

All ROIs were related to the horizontal–vertical orientation of stereoscopic surfaces. Among these areas, the dorsal areas V3A and V7, the higher ventral area LOC, and the parietal areas VIPS and POIPS showed a more generalized orientation representation irrespective of the sign of curvature of surface.

## Ethics Statement

This study was carried out in accordance with the recommendations of Human Research Ethics Committee of Kochi University of Technology with written informed consent from all subjects. All subjects gave written informed consent in accordance with the Declaration of Helsinki. The protocol was approved by the Human Research Ethics Committee of Kochi University of Technology.

## Author Contributions

ZL and HS designed the study and collected the data together. ZL contributed to data analysis and wrote the manuscript. HS supervised data analysis and revised the manuscript. All authors approved the final manuscript.

## Conflict of Interest Statement

The authors declare that the research was conducted in the absence of any commercial or financial relationships that could be construed as a potential conflict of interest.

## References

[B2] Alizadeh S. Jamalabadi H. Schönauer M. Leibold C. Gais S. (2017). Decoding cognitive concepts from neuroimaging data using multivariate pattern analysis. Neuroimage 159, 449–458. 10.1016/j.neuroimage.2017.07.058 28765057

[B1] Alizadeh A. M. Van Dromme I. C. Janssen P. (2018). Single-cell responses to three-dimensional structure in a functionally defined patch in macaque area TEO. J. Neurophysiol. 120, 2806–2818. 10.1152/jn.00198.2018 30230993 PMC6337039

[B3] Anzai A. Chowdhury S. A. DeAngelis G. C. (2011). Coding of stereoscopic depth information in visual areas V3 and V3A. J. Neurosci. 31, 10270–10282. 10.1523/JNEUROSCI.5956-10.2011 21753004 PMC3143190

[B4] Ban H. Welchman A. E. (2015). fMRI analysis-by-synthesis reveals a dorsal hierarchy that extracts surface slant. J. Neurosci. 35, 9823–9835. 10.1523/JNEUROSCI.1255-15.2015 26156985 PMC4495240

[B5] Chandrasekaran C. Canon V. Dahmen J. C. Kourtzi Z. Welchman A. E. (2007). Neural correlates of disparity-defined shape discrimination in the human brain. J. Neurophysiol. 97, 1553–1565. 10.1152/jn.01074.2006 17151220

[B6] Cumming B. G. DeAngelis G. C. (2001). The physiology of stereopsis. Annu. Rev. Neurosci. 24, 203–238. 10.1146/annurev.neuro.24.1.203 11283310

[B7] Cumming B. G. Parker A. J. (1997). Responses of primary visual cortical neurons to binocular disparity without depth perception. Nature 389, 280–283. 10.1038/38487 9305841

[B8] DeYoe E. A. Carman G. J. Bandettini P. Glickman S. Wieser J. Cox R.. (1996). Mapping striate and extrastriate visual areas in human cerebral cortex. Proc. Natl. Acad. Sci. U S A 93, 2382–2386. 10.1073/pnas.93.6.2382 8637882 PMC39805

[B9] Dövencioğlu D. Ban H. Schofield A. J. Welchman A. E. (2013). Perceptual integration for qualitatively different 3-D cues in the human brain. J. Cogn. Neurosci. 25, 1527–1541. 10.1162/jocn_a_00417 23647559 PMC3785137

[B10] Dupont P. De Bruyn B. Vandenberghe R. Rosier A. M. Michiels J. Marhal G.. (1997). The kinetic occipital region in human visual cortex. Cereb. Cortex 7, 283–292. 10.1093/cercor/7.3.283 9143447

[B11] Fischl B. (2012). FreeSurfer. Neuroimage 62, 774–781. 10.1016/j.neuroimage.2012.01.021 22248573 PMC3685476

[B12] Freud E. Ganel T. Shelef I. Hammer M. D. Avidan G. Behrmann M. (2017). Three-dimensional representations of objects in dorsal cortex are dissociable from those in ventral cortex. Cereb. Cortex 27, 422–434. 10.1093/cercor/bhv229 26483400 PMC13100970

[B14] Georgieva S. Peeters R. Kolster H. Todd J. T. Orban G. A. (2009). The processing of three-dimensional shape from disparity in the human brain. J. Neurosci. 29, 727–742. 10.1523/JNEUROSCI.4753-08.2009 19158299 PMC6665151

[B13] Georgieva S. S. Todd J. T. Peeters R. Orban G. A. (2008). The extraction of 3D shape from texture and shading in the human brain. Cereb. Cortex 18, 2416–2438. 10.1093/cercor/bhn002 18281304 PMC2536698

[B15] Goncalves N. R. Ban H. Sánchez-Panchuelo R. M. Francis S. T. Schluppeck D. Welchman A. E. (2015). 7 tesla FMRI reveals systematic functional organization for binocular disparity in dorsal visual cortex. J. Neurosci. 35, 3056–3072. 10.1523/JNEUROSCI.3047-14.2015 25698743 PMC4331627

[B16] Goodale M. A. Milner A. D. (1992). Separate visual pathways for perception and action. Trends Neurosci. 15, 20–25. 10.1016/0166-2236(92)90344-8 1374953

[B17] Grossberg S. Srinisan K. Yazdanbakhsh A. (2011). On the road to invariant object recognition: how cortical area V2 transforms absolute to relative disparity during 3D vision. Neural Netw. 24, 686–692. 10.1016/j.neunet.2011.03.021 21507610

[B18] Horikawa T. Kamitani Y. (2017). Generic decoding of seen and imaged objects using hierarchical visual features. Nat. Commun. 8:15037. 10.1038/ncomms15037 28530228 PMC5458127

[B19] Hubel D. H. Wiesel T. N. Yeagle E. M. Lafer-Sousa R. Conway B. R. (2015). Binocular stereoscopy in visual areas V-2, V-3, and V-3A of the macaque monkey. Cereb. Cortex 25, 959–971. 10.1093/cercor/bht288 24122139 PMC4074265

[B22] Janssen P. Vogels R. Liu Y. Orban G. A. (2001). Macaque inferior temporal neurons are selective for three-dimensional boundaries and surfaces. J. Neurosci. 21, 9419–9429. 10.1523/JNEUROSCI.21-23-09419.2001 11717375 PMC6763913

[B20] Janssen P. Vogels R. Orban G. A. (1999). Macaque inferior temporal neurons are selective for disparity-defined three-dimensional shapes. Proc. Natl. Acad. Sci. U S A 96, 8217–8222. 10.1073/pnas.96.14.8217 10393975 PMC22215

[B21] Janssen P. Vogels R. Orban G. A. (2000). Three-dimensional shape coding in inferior temporal cortex. Neuron 27, 385–397. 10.1016/s0896-6273(00)00045-3 10985357

[B23] Johnston A. Passmore P. J. (1994). Independent encoding of surface orientation and surface curvature. Vision Res. 34, 3005–3012. 10.1016/0042-6989(94)90273-9 7975335

[B24] Koenderink J. J. Doorn A. J. V. (1992). Surface shape and curvature scales. Image Vis. Comput. 10, 557–564. 10.1016/0262-8856(92)90076-f

[B25] Konen C. S. Kastner S. (2008). Two hierarchically organized neural systems for object information in human visual cortex. Nat. Neurosci. 11, 224–231. 10.1038/nn2036 18193041

[B26] Kourtzi Z. Kanwisher N. (2000). Cortical regions involved in perceiving object shape. J. Neurosci. 20, 3310–3318. 10.1523/JNEUROSCI.20-09-03310.2000 10777794 PMC6773111

[B27] Kourtzi Z. Kanwisher N. (2001). Representation of perceived object shape by the human lateral occipital complex. Science 293, 1506–1509. 10.1126/science.1061133 11520991

[B28] Lehky S. R. Sereno A. B. (2007). Comparison of shape encoding in primate dorsal and ventral visual pathways. J. Neurophysiol. 97, 307–319. 10.1152/jn.00168.2006 17021033

[B29] Logothetis N. K. Pauls J. Augath M. Trinath T. Oeltermann A. (2001). Neurophysiological investigation of the basis of the fMRI signal. Nature 412, 150–157. 10.1038/35084005 11449264

[B30] Marr D. Poggio T. (1976). Cooperative computation of stereo disparity. Science 194, 283–287. 10.1126/science.968482 968482

[B31] Mishkin M. Ungerleider L. G. (1982). Contribution of striate inputs to the visuospatial functions of parieto-preoccipital cortex in monkeys. Behav. Brain Res. 6, 57–77. 10.1016/0166-4328(82)90081-x 7126325

[B32] Murphy A. P. Ban H. Welchman A. E. (2013). Integration of texture and disparity cues to surface slant in dorsal visual cortex. J. Neurophysiol. 110, 190–203. 10.1152/jn.01055.2012 23576705 PMC3727040

[B33] Naganuma T. Nose I. Inoue K. Takemoto A. Katsuyama N. Taira M. (2005). Information processing of geometrical features of a surface based on binocular disparity cues: an fMRI study. Neurosci. Res. 51, 147–155. 10.1016/j.neures.2004.10.009 15681032

[B34] Neri P. (2005). A stereoscopic look at visual cortex. J. Neurophysiol. 93, 1823–1826. 10.1152/jn.01068.2004 15774707

[B35] Neri P. Bridge H. Heeger D. J. (2004). Stereoscopic processing of absolute and relative disparity in human visual cortex. J. Neurophysiol. 92, 1880–1891. 10.1152/jn.01042.2003 15331652

[B36] Nguyenkim J. D. DeAngelis G. C. (2003). Disparity-based coding of three-dimensional surface orientation by macaque middle temporal neurons. J. Neurosci. 23, 7117–7128. 10.1523/JNEUROSCI.23-18-07117.2003 12904472 PMC6740667

[B37] Orban G. A. Claeys K. Nelissen K. Smans R. Sunaert S. Todd J. T.. (2006a). Mapping the parietal cortex of human and non-human primates. Neuropsychologia 44, 2647–2667. 10.1016/j.neuropsychologia.2005.11.001 16343560

[B38] Orban G. A. Janssen P. Vogels R. (2006b). Extracting 3D structure from disparity. Trends Neursci. 29, 466–473. 10.1016/j.tins.2006.06.012 16842865

[B39] Orban G. A. Sunaert S. Todd J. T. Van Hecke P. Marchal G. (1999). Human cortical regions involved in extracting depth from motion. Neuron 24, 929–940. 10.1016/s0896-6273(00)81040-5 10624956

[B40] Paradis A. L. Cornilleau-Pérès V. Droulez J. Van De Moortele P. E. Lobel E. Berthoz A.. (2000). Visual perception of motion and 3-D structure from motion: an fMRI study. Cereb. Cortex 10, 772–783. 10.1093/cercor/10.8.772 10920049

[B41] Parker A. J. (2007). Binocular depth perception and the cerebral cortex. Nat. Rev. Neurosci. 8, 379–391. 10.1038/nrn2131 17453018

[B42] Patten M. L. Welchman A. E. (2015). fMRI activity in posterior parietal cortex relates to the perceptual use of binocular disparity for both signal-in-noise and feature difference tasks. PLoS One 10:e0140696. 10.1371/journal.pone.0140696 26529314 PMC4631361

[B43] Preston T. J. Kourtze Z. Welchman A. E. (2009). Adaptive estimation of three-dimensional structure in the human brain. J. Neurosci. 29, 1688–1698. 10.1523/JNEUROSCI.5021-08.2009 19211876 PMC6666271

[B44] Preston T. J. Li S. Kourtzi Z. Welchman A. E. (2008). Multivoxel pattern selectivity for perceptual relevant binocular disparities in the human brain. J. Neurosci. 28, 11315–11327. 10.1523/JNEUROSCI.2728-08.2008 18971473 PMC6671500

[B45] Roe A. W. Parker A. J. Borm R. T. DeAngelis G. C. (2007). Disparity channels in early vision. J. Neurosci. 27, 11820–11831. 10.1523/JNEUROSCI.4164-07.2007 17978018 PMC2376798

[B46] Rosenberg A. Cowan N. J. Angelaki D. E. (2013). The visual representation of 3D object orientation in parietal cortex. J. Neurosci. 33, 19352–19361. 10.1523/JNEUROSCI.3174-13.2013 24305830 PMC3850047

[B47] Sakata H. Taira M. Kusunoki M. Murata A. Tanaka Y. Tsutsui K. (1998). Neural coding of 3D features of objects for hand action in the parietal cortex of the monkey. Philos. Trans. R. Soc. Lond. B Biol. Sci. 29, 1363–1373. 10.1098/rstb.1998.0290 9770229 PMC1692338

[B48] Schiller P. H. Logothetis N. K. Charles E. S. (1990). Role of the color-opponent and broad-band channels in vision. Vis. Neurosci. 29, 321–345. 10.1017/s0952523800000420 2265148

[B50] Sereno M. I. Dale A. M. Reppas J. B. Kwong K. K. Belliveau J. W. Brady T. J.. (1995). Borders of multiple visual areas in human revealed by functional magnetic resonance imaging. Science 268, 889–893. 10.1126/science.7754376 7754376

[B49] Sereno M. E. Trinath T. Augath M. Logothetis N. K. (2002). Three-dimensional shape representation in monkey cortex. Neuron 14, 635–652. 10.1016/s0896-6273(02)00598-6 11856536

[B51] Shikata E. Hamzei F. Glauche V. Knab R. Dettmers C. Weiller C.. (2001). Surface orientation discrimination activates caudal and anterior intraparietal sulcus in humans: an event-related fMRI study. J. Neurophysiol. 85, 1309–1314. 10.1152/jn.2001.85.3.1309 11247999

[B52] Shikata E. Tanaka Y. Nakamura H. Taira M. Sakata H. (1996). Selectivity of the parietal visual neurones in 3D orientation of surface of stereoscopic stimuli. Neuroreport 7, 2389–2394. 10.1097/00001756-199610020-00022 8951858

[B53] Sim E. J. Helbig H. B. Graf M. Kiefer M. (2015). When action observation facilitates visual perception: activation in visuo-motor areas contributes to object recognition. Cereb. Cortex 25, 2907–2918. 10.1093/cercor/bhu087 24794918

[B54] Taira M. Nose I. Inoue K. Tsutsui K. (2001). Cortical areas related to attention to 3D surface structures based on shading: an fMRI study. Neuroimage 14, 959–966. 10.1006/nimg.2001.0895 11697928

[B55] Taira M. Tsutsui K. I. Jiang M. Yara K. Sakata H. (2000). Parietal neurons represent surface orientation from the gradient of binocular disparity. J. Neurophysiol. 83, 3140–3146. 10.1152/jn.2000.83.5.3140 10805708

[B56] Takemura H. Rokem A. Winawer J. Yeatman J. D. Wandell B. A. Pestilli F. (2016). A major human white matter pathway between dorsal and ventral visual cortex. Cereb. Cortex 26, 2205–2214. 10.1093/cercor/bhv064 25828567 PMC4830295

[B57] Thomas O. M. Cumming B. G. Parker A. J. (2002). A specialization for relative disparity in V2. Nat. Neurosci. 5, 472–478. 10.1038/nn837 11967544

[B58] Tootell R. B. H. Hadjikhani N. Hall E. K. Marrett S. Vanduffel W. Vaughan J. T.. (1998). The retinotopy of visual spatial attention. Neuron 21, 1409–1422. 10.1016/s0896-6273(00)80659-5 9883733

[B60] Tsutsui K. Jiang M. Yara K. Sakata H. Taira M. (2001). Integration of perspective and disparity cues in surface-orientation-selective neurons of area CIP. J. Neurophysiol. 86, 2856–2867. 10.1152/jn.2001.86.6.2856 11731542

[B59] Tsutsui K. Sakata H. Naganuma T. Taira M. (2002). Neural correlates for perception of 3D surface orientation from texture gradient. Science 298, 409–412. 10.1126/science.1074128 12376700

[B61] Tyler C. W. Likova L. T. Chen C. C. Kontsevich L. L. Schira M. M. Wade A. R. (2005). Extended concepts of occipital retinotopy. Curr. Med. Imaging Rev. 1, 319–329. 10.2174/157340505774574772

[B62] Uka T. DeAngelis G. C. (2006). Linking neural representation to function in stereoscopic depth perception: roles of the middle temporal area in coarse versus fine disparity discrimination. J. Neurosci. 26, 6791–6802. 10.1523/JNEUROSCI.5435-05.2006 16793886 PMC1994558

[B63] Van Dromme I. C. Premereur E. Verhoef B. E. Vanduffel W. Janssen P. (2016). Posterior parietal cortex drives inferotemporal activation during three-dimensional object vision. PLoS Biol. 14:e1002445. 10.1371/journal.pbio.1002445 27082854 PMC4833303

[B64] Vanduffel W. Fize D. Peuskens H. Denys K. Sunaert S. Todd J. T.. (2002). Extracting 3D from motion: differences in human and monkey intraparietal cortex. Science 298, 413–415. 10.1126/science.1073574 12376701

[B65] Warnking J. Dojat M. Guérin-Dugué A. Delon-Martin C. Olympiesff S. Richard N.. (2002). fMRI retinotopic mapping—step by step. Neuroimage 17, 1665–1683. 10.1006/nimg.2002.1304 12498741

[B66] Woods R. P. Grafton S. T. Holmes C. J. Cherry S. R. Mazziotta J. C. (1998). Automated image registration: I. General methods and intrasubject, intramodality validation. J. Comput. Assist. Tomogr. 22, 139–152. 10.1097/00004728-199801000-00027 9448779

[B67] Yeatman J. D. Weiner K. S. Pestili F. Pokem A. Mezer A. Wandell B. A. (2014). The vertical occipital fasciculus: a century of countroversy resolved by *in vivo* measurements. Proc. Natl. Acad. Sci. U S A 111, E5214–E5223. 10.1073/pnas.1418503111 25404310 PMC4260539

[B68] Zeki S. M. (1978). Functional specialization in the visual cortex of the rhesus monkey. Nature 274, 423–428. 10.1038/274423a0 97565

[B69] Zeki S. Perry R. J. Bartels A. (2003). The processing of kinetic contours in the brain. Cereb. Cortex 13, 189–202. 10.1093/cercor/13.2.189 12507950

[B70] Zeki S. Watson J. D. Lueck C. J. Friston K. J. Kennard C. Frackowiak R. S. (1991). A direct demonstration of functional specialization in human visual cortex. J. Neurosci. 11, 641–649. 10.1523/JNEUROSCI.11-03-00641.1991 2002358 PMC6575357

